# Association of Maxillary Sinus Reactions and Periapical Pathology in the Maxillary Posterior Teeth: Evaluation Using Cone Beam Computed Tomography

**DOI:** 10.1590/0103-644020245973

**Published:** 2024-12-06

**Authors:** Alba Elizabeth do Nascimento Gomes, Carlos Eduardo da Silveira Bueno, Alexandre Sigrist De Martin, Carolina Pessoa Stringheta, Carlos Eduardo Fontana, Daniel Guimarães Pedro Rocha, Ana Grasiela Limoeiro, Wayne Martins Nascimento, Marilia Fagury Videira Marceliano-Alves, Rina Andrea Pelegrine

**Affiliations:** 1 Faculdade São Leopoldo Mandic, Instituto de Pesquisas São Leopoldo Mandic, Departament of Endodontics, Campinas, SP, Brazil.; 2 PUC Campinas, Center of Life Sciences, Programa de pós-graduação em Ciências da Saúde, Campinas, SP, Brazil.; 3 Department of Dentistry, Endodontics and Dental Materials, Bauru Dental School, University of Sao Paulo, Bauru, Brazil.; 4 Department of Endodontics and Dental Reseach, Iguaçu University, Nova Iguaçu, Brazil.; 5 KU Leuven (University of Leuven), Department of Oral Health Sciences, BIOMAT - Biomaterials Research group & UZ Leuven (University Hospitals Leuven), Dentistry, Leuven, Belgium.; 6 Laboratory of Orofacial Pathologies, Imaging and Biotherapies, School of Dentistry, Laboratoire d’Excellence INFLAMEX, Université Paris Cité, URP 2496, Montrouge. France.

**Keywords:** Cone beam computed tomography, Maxillary sinusitis, Periapical diseases

## Abstract

This study aimed to investigate the association between maxillary sinus reactions and periradicular pathology in maxillary posterior teeth using cone-beam computed tomography. The maxillary posterior images of 395 teeth were examined for the presence of periradicular pathology, and the maxillary sinus disease. The proximity between the root apex and the cortical bone of the maxillary sinus using the linear measurement (mm) tool was used (RadiAnt, DICOM viewer, Poznan, Poland). The frequency found from maxillary sinus assessment was subjected to the Kolmogorov-Smirnov normality test, the Kruskal-Wallis test, the chi-square test with Bonferroni correction and a logistic regression analysis. A significance level of 5% was assumed. About 46.6% of the teeth showed a periapical pathology associated with an inflammatory reaction of the maxillary sinus. Among them, thickening of the maxillary sinus mucosa > 3 mm is the most common finding (59.3%). There was no difference between the types of maxillary sinus reactions and the variables studied (p >0.05). Periapical lesions on palatal roots were 2.17 times more likely to develop an inflammatory reaction than other roots (p < 0.05). Women were 2.04 times less likely to develop maxillary changes than men (p < 0.05). The distance between apex and floor and the presence or absence of endodontic treatment were not significantly associated with sinusitis. Periapical pathology could be related to maxillary inflammatory reaction of the sinus.

## Resumo

estudo teve como objetivo investigar a relação entre doença sinusal e patologia perirradicular nos dentes posteriores superiores por meio de tomografia computadorizada de feixe cônico. As imagens posteriores da maxila de 395 dentes foram examinadas para patologia perirradicular, o seio maxilar para doença sinusal e a proximidade entre o ápice da raiz e o osso cortical do seio maxilar. Os dados foram submetidos ao teste de normalidade de Kolmogorov-Smirnov, teste de Kruskal-Wallis, teste qui-quadrado com correção de Bonferroni e análise de regressão logística. Foi assumido um nível de significância de 5%.nCerca de 46,6% dos dentes apresentavam patologia periapical envolvida com sinusopatia (46,6%). Dentre eles, o espessamento da mucosa sinusal > 3mm é o achado mais comum (59,3%). Não houve diferença entre os tipos de sinusopatias e as variáveis estudadas (p >0,05). Lesões periapicais nas raízes palatinas tiveram 2,17 vezes mais probabilidade de desenvolver doença sinusal do que outras raízes (p < 0,05). As mulheres tiveram 2,04 vezes menos probabilidade do que os homens de desenvolver sinusite (p<0,05). A distância ápice-chão e a presença ou ausência de tratamento endodôntico não foram significativamente associadas à sinusite. A patologia periapical pode estar relacionada a uma doença sinusal.

## Introduction

Sinusitis is an inflammation of the Schneiderian membrane, a mucous membrane of the paranasal sinuses, usually caused by allergies or respiratory infections. It is known that other conditions besides respiratory diseases, such as odontogenic infections, can lead to sinusitis, and the number of cases seems to have increased in recent decades ^1^ ^,^ ^2^.

Periapical lesions in the maxillary posterior region are of clinical importance due to the proximity of the root apices to the maxillary sinus, so odontogenic infections can lead to inflammatory changes in the maxillary sinus mucosa and the subsequent development of sinusitis ^1^ ^,^ ^3^. The continuous expansion and pneumatization of the maxillary sinus that occurs in some patients throughout life can sometimes lead to an anatomical condition in which only the mucoperiosteum separates the tips of the teeth from the maxillary sinuses. This condition may favor the spread of odontogenic infections to the maxillary sinus ^4^.

Odontogenic sinusitis is usually polymicrobial, with anaerobic species originating from the oral cavity and upper respiratory tract predominating in 66.7% of cases ^5^. It is thought that microorganisms present in periapical infections may migrate into the maxillary sinus and involve the Schneiderian membrane, increasing the risk of an inflammatory reaction, and that changes in this membrane can then be detected in imaging studies ^3^.

The risk factors for maxillary sinusitis with an endodontic origin in the posterior maxilla are widespread. Some factors may be related, such as: inadequate treatment, presence of periapical periodontitis and the position of the root apex in contact with the maxillary sinus ^3^ ^,^ ^5^. The anatomical proximity between the radicular apexes (especially in molars) and the maxillary sinus cortical bone has the potential to spread infection into the maxillary sinuses, that can lead to sinus infection of odontogenic origin requiring multiprofessional assistance ^3^. In this case, it is important to evaluate the anatomical aspects of the maxillary sinus as well as the frequency of sinus changes when endodontic infection is present ^2^ ^,^ ^3^ ^,^ ^5^.

Cone-beam computed tomography (CBCT) is an important method to evaluate these maxillary sinus changes and, in combination with clinical signs such as facial pain, toothache, nasal secretion and/or obstruction, facial discomfort, and bad odor, provides information for the correct diagnosis ^6^. Although the periapical radiograph is the routine examination in endodontics, it is not the appropriate tool to assess the anatomical relationships between the maxillary molars and the maxillary sinus floor as it provides a two-dimensional image and does not allow visualization of the entire maxillary sinus volume ^7^.

According to the above, the aim of this study was to evaluate the association between the inflammatory response in the maxilla and periradicular pathologies of the maxillary posterior teeth using CBCT. To this end, the following factors were evaluated: (a) the gender ratio relationship and the presence of maxillary sinus disease; (b) the teeth or roots most associated with maxillary sinus disease; (c) the distance between the root apex and the floor of the maxillary sinus in the presence of maxillary sinus disease; (d) the association between the maxillary sinus response and endodontically treated teeth with apical periodontitis.

The null hypotheses were: 1. Association between maxillary sinus reaction and gender; 2. The tooth type influences the occurrence of maxillary sinus reaction; 3. root type influences the sinus inflammatory reaction; 4. distance between the root apex and the floor of the maxillary sinus influences the occurrence of maxillary sinus reaction; 5. endodontically treated tooth with apical periodontitis influences inflammatory reaction of the maxillary sinus.

## Materials and Methods

This study was conducted in accordance with the requirements of resolution 196/96 of the National Health Council. The project was submitted to and approved by the local research Ethics Committee (n. 4.601.417).

The sample was calculated based on the study by Nascimento et al. ^8^, who evaluated 400 CBCT scans. A retrospective observational study was performed with 395 exams from 2000 CBCT exams data acquired between January 2018 and December 2020 using anICAT Classic (Imaging Sciences International, Hatfield, USA) with a field of view (FOV) of 16 cm x 6 cm ^9^, 120 KVp, 5mA, and exposure time of 40s. The image volume was reconstructed with isotropic isometric voxels of 0.25 x 0.25 x 0.25 mm. The images in DICOM format (Digital Imaging and Communication in Medicine) were processed, interpreted and measured using 3D Dicom E-Vol DX software - version 5.0.1.39 (CDT, Consultoria, Desenvolvimento e Ensino Educacional, Dourados, Brazil). An endodontic specialist performed all examinations.

Inclusion criteria included: exams from individuals between 18 and 80 years of age of both genders; teeth with periradicular pathologies; exams from the maxillary region using the ICAT Classic. The exclusion criteria were exams with low quality; patients with mixed dentition, syndromic disease, edentulous, presence of developmental lesions in the maxilla, patients with dental implants in the posterior maxilla or zygomatic region, patients who have undergone orthognathic surgery, posterior teeth with cast metal core that can produce artifacts.

After analyzing the entire CBCT volume, the sagittal tomographic slices were selected to better visualize the relationship between the periapex of the roots and the inner cortex of the maxillary sinus adjacent to this region. The distance between the periapex between the root of the maxillary molar and the inner cortex of the maxillary sinus was emphasized, and the linear measurement tool (RadiAnt, DICOM viewer, Poznan, Poland) was used to quantify the distance (mm).

**Figure 1:**
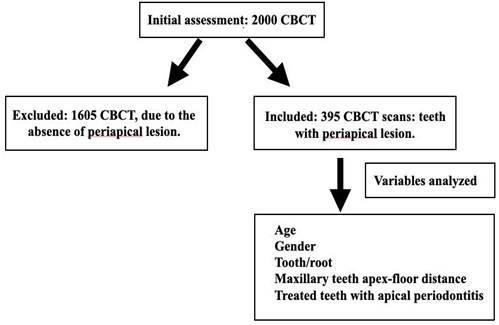
Flowchart showing the audit selection steps and variables analyzed.

The maxillary sinus and periapical pathology were assessed in both sagittal and coronal slices. However, the measurements were only performed on sagittal slices. The assessment of maxillary sinus pathology was performed from the cortex of the maxillary sinus floor to the upper point of the maxillary sinus (figure 1).

The maxillary molars and premolars with periradicular pathologies (hypodense area around the root apex or thickening of the periodontal ligament of 0.5 mm or more) were considered for the evaluation. The maxillary sinuses were examined for the presence of maxillary sinus disease. Since not all roots have the same spatial relationship to the maxillary sinus, the root and/or periapical lesion closest to the maxillary sinus floor was considered.

**Figure 2:**
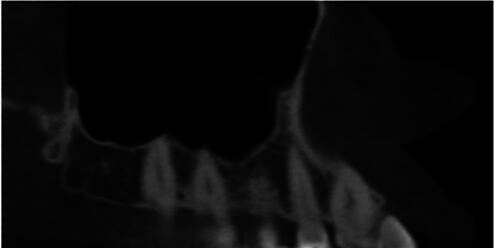
Sagittal section of a tomographic image of the normal maxillary sinus (no alteration).

**Figure 3:**
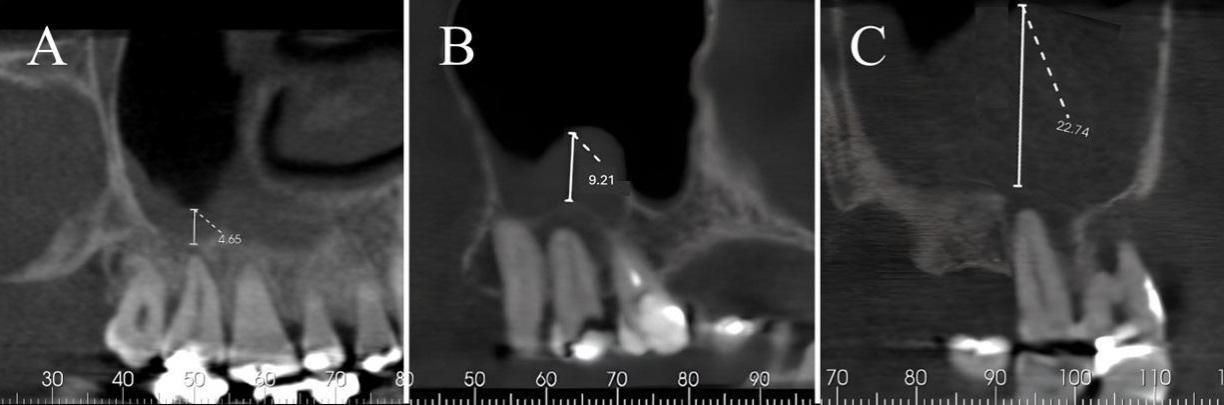
Sagittal section of cone beam tomography images showing the measurements of sinus diseases. A) Thickening of the sinus mucosa > 3mm. B) Partial sinus veiling > 6mm. C) Total sinus veiling > 12mm.

**Figure 4:**
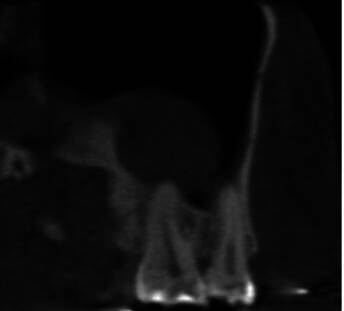
Sagittal section of tomographic image of the mucus retention phenomenon.

The adopted criteria for the maxillary sinus disease classification were those proposed by Cure et al.,^10^. Maxillary sinuses with a mucosal thickness of less than 3 mm at the floor were considered healthy (figure 2). Thickening of the maxillary sinus mucosa with 3 mm or more; partial maxillary sinus obstruction when the mucosa measured 6 mm or more; total maxillary sinus obstruction when the mucosa measured 12 mm or occupied the entire length of the maxillary sinus (figure 3). Mucus retention phenomenon when a hyperdense, dome-shaped image was present inside the maxillary sinus (figure 4).

Data obtained were analyzed using descriptive and inferential statistics in IBM SPSS software (26.0, IBM Corporation, Armonk, USA). For the quantitative variables, the normality of the distribution was assessed using the Kolmogorov-Smirnov test, which yielded a p< 0.05, indicating a non normal distribution of the data. Next, the distribution of the sample in the categories of the variables of interest was evaluated, and the frequencies of the distribution of the sample in each of the variables of interest were compared for each of the diagnosed types of maxillary sinus reaction using the Kruskal-Wallis test and the chi-square test with Bonferroni correction. Finally, the variables of interest were evaluated as predictors of the outcome "maxillary sinus reaction" by logistic regression analysis. Based on the univariate analysis, the variables of interest were selected for fitting the multiple models, and based on this selection, the backward method was used to fit the final explanatory model for the diagnosis of sinus inflammatory reaction from the variables selected for the present study. A significance level of 5% was assumed for all analyzes.

## Results

The sample consisted of 395 CBCT scans and a single tooth per scan, presenting periapical pathology, was analyzed. The mean age of the patients included in the study was 46.57 (±13.20) years and a median of 47.00 (p25=38.00; p75=56.00), with 175 (44.3%) men and 220 (55.7%) women. Table 1 contains the distribution of the variables analyzed (tooth, root, presence or absence of endodontic treatment and type of maxillary sinus reaction) and the frequency of these variables, with a prevalence of 46.6% of maxillary sinus inflammatory reaction of the sinus in teeth with pathologic periapical sinuses.

The most prevalent sinus inflammatory reaction was the mucosal thickening > 3 mm (59.3%), followed by partial sinus obstruction > 6 mm (22.8%), total obstruction > 12 mm (16.3%), and mucus retention (1.6%).

Among the periapical pathologies, the most common teeth were the maxillary first molar (16 - 28.4% and 26 - 30.9%), followed by the second molar (17 - 15.7% and 27 - 11.1%) and second premolar (15 - 7.1% and 25 -5.8%). The roots were: mesiobuccal (51.1%), followed by palatal (15.3%), distobuccal (15.2%) and buccal roots of premolars (13.4%). Of the teeth examined, 40.8% had undergone previous endodontic treatment (table 1).

**Table 1:** Frequency distribution of the sample in each of the categories of variables of interest.

Variable	n (%)
Tooth associated with periapical pathology	
14	2 (0,5%)
15	28 (7,1%)
16	112 (28,4%)
17	62 (15,7%)
18	2 (0,5%)
25	23 (5,8%)
26	122 (30,9%)
27	44 (11,1%)
Root related to periapical pathology	
Vestibular	53 (13,4%)
Mesiobuccal	202 (51,1%)
Disto-vestibular	60 (15,2%)
Palatine	80 (20,3%)
Endodontic treatment	
Yes	161 (40,8%)
No	234 (59,2%)
Sinus disease	
Yes	184 (46,6%)
No	211 (53,4%)
Type of sinus disease	
Mucosal thickening > 3mm	109 (59,3%)
Partial veiling of the maxillary sinus > 6mm	42 (22,8%)
Total veiling of the maxillary sinus > 12mm	30 (16,3%)
Mucus retention	3 (1,6%)

According to the Kruskal-Wallis test and the chi-square test with Bonferroni correction, no statistically significant differences were found between the types of maxillary sinus inflammatory response and the studied variables (age, gender, tooth, root associated with periapical pathology, endodontic treatment, and apex-to-floor length), indicating that there was no association between the variables of interest and the types of maxillary sinus inflammatory response (p > 0.05) (table 2).

**Table 2:** Evaluation and comparison between the researched variables and the types of sinus diseases evaluated.

Variables	Sinus diseases				p
	Mucosal thickening > 3mm (n= 109)	Partial veiling of the maxillary sinus > 6mm (n=42)	Total veiling of the maxillary sinus > 12mm (n=30)	Mucus retention (n=3)	
Age Median (p25, p75)					
	49,00 (38,00; 58,00)	49,50 (36,75; 59,25)	48,50 (37,50; 58,25)	38,00 ( - )	0,853 *
Gender					
Male	51 (46,8%)	29 (69,0%)	17 (56,7%)	1 (33,3%)	0,063 **
Female	58 (53,2%)	13 (31,0%)	13 (43,3%)	2 (66,7%)	
Tooth					
14	1 (0,9%)	0 (0,0%)	0 (0,0%)	0 (0,0%)	0,789 **
15	11 (10,2%)	1 (2,4%)	1 (3,3%)	0 (0,0%)	
16	30 (27,5%)	13 (31,0%)	7 (23,3%)	0 (0,0%)	
17	13 (11,9%)	7 (16,7%)	8 (26,8%)	2 (66,7%)	
18	0 (0,0%)	0 (0,0%)	1 (3,3%)	0 (0,0%)	
25	5 (4,6%)	4 (9,5%)	0 (0,0%)	0 (0,0%)	
26	36 (33,0%)	10 (23,8%)	9 (30,0%)	1 (33,3%)	
27	13 (11,9%)	7 (16,6%)	4 (13,3%)	0 (0,0%)	
Root related to periapical pathology					
Vestibular	17 (15,6%)	5 (11,9%)	1 (3,3%)	0 (0,0%)	0,643 **
Mesiobuccal	42 (38,5%)	21 (50,0%)	17 (56,7%)	2 (66,7%)	
Disto-vestibular	18 (16,5%)	7 (16,7%)	5 (16,7%)	0 (0,0%)	
Palatine	32 (29,4%)	9 (21,4%)	7 (23,3%)	1 (33,3%)	
Endodontic treatment					
Yes	42 (38,5%)	15 (35,7%)	12 (40,0%)	0 (0,0%)	0,643 **
No	67 (61,5%)	27 (64,3%)	18 (60,0%)	3 (100,0%)	
Apex-floor distance Median (p25, p75)					
	1,66 (0,94; 2,23)	1,58 (1,00; 2,15)	1,48 (0,00; 2,21)	0,00 (-)	0,113 *

* Kruskal-Wallis test; ** Chi-square test with Bonferroni correction. Significance level=5%.

Logistic regression analysis was used to test the probability of occurrence of maxillary sinus inflammatory response as an outcome related to the variables of interest. Only gender and root variables were significantly associated with maxillary sinusitis (p< 0.05). Women were 2.04 times less likely than men to have maxillary sinusitis (table 3).

In this study, the palatine root had a 2.17 times more likely to develop maxillary sinusitis than the others (p<0.05). The outcome of maxillary sinusitis was not significantly associated with the type of teeth examined, the absence of endodontic treatment or the distance between the maxillary sinus cortical bone and the root apices (table 3).

**Table 3:** Evaluation of variables of interest in relation to the outcome “sinus disease” using unadjusted and adjusted logistic regression models.

Variable	sinus disease		unadjusted		adjusted	
	No	Yes	OR (IC95%)		OR (IC95%)	
Age median (p25; p75)						
	45,00 (38,00; 53,00)	49,00 (38,00; 58,00)	0,134	1,01 (0,99-1,02)	-	-
Gender						
Male	77 (36,5%)	98 (53,3%)	-	1,00	-	1,00
Female	134 (63,5%)	86 (46,7%)	0,001	0,50 (0,33-0,75)	0,001	0,49(0,32-0,74)
Tooth						
14	1 (0,5%)	1 (0,5%)	-	1,00	-	-
15	15 (7,0%)	13 (7,2%)	0,922	0,86(0,04-15,27)	-	-
16	62 (29,4%)	50 (27,2%)	0,880	0,80(0,04-13,21)	-	-
17	32 (15,2%)	30 (16,3%)	0,964	0,93(0,05-15,66)	-	-
18	1 (0,5%)	1 (0,5%)	1,000	1,00(0,02-50,39)	-	-
25	14 (6,6%)	9 (4,9%)	0,765	0,64(0,03-11,63)	-	-
26	66 (31,3%)	56 (30,4%)	0,908	0,84(0,05-13,87)	-	-
27	20 (9,5%)	24 (13,0%)	0,900	1,20(0,07-20,42)	-	-
Root related to periapical pathology						
Vestibular	30 (14,2%)	23(12,5%)	-	1,00	-	1,00
Mesiobuccal	120 (56,9%)	82 (44,6%)	0,712	0,89 (0,48-1,64)	0,795	0,92(0,49-1,71)
Disto-vestibular	30 (14,2%)	30 (16,3%)	0,483	1,30 (0,62-2,74)	0,378	1,40 (0,66-2,98)
Palatine	31 (14,7%)	49 (26,6%)	0,044	2,06 (1,01-4,17)	0,034	2,17 (1,06-4,44)
Endodontic treatment						
Yes	92 (43,6%)	69 (37,5%)	-	1,00	-	-
No	119 (56,4%)	115 (62,5%)	0,219	1,28 (0,86-1,93)	-	-
Apex-floor distance						
	1,37(0,89; 1,91)	1,55(0,88;2,12)	0,724	1,03 (0,87-1,21)	-	-

p25 = 25th percentile; p75 = 75th percentile. OR = Odds Ratio. 95% CI = 95% confidence interval. Logistic regression. Significance level = 5%.

## Discussion

The risk factors for maxillary sinusitis of endodontic origin in maxillary posterior teeth are high and can be associated with endodontic treatment, apical periodontitis and the position of the root apex in contact with the floor of the maxillary sinus ^1^ ^,^ ^2^ ^,^ ^3^ ^,^ ^4^. The frequency of occurrence of these factors was evaluated using dynamic navigation and a new filter of CBCT ^11^. The anatomical proximity between the root apices (especially the molars) and the maxillary sinus cortices makes periradicular pathology a potential source for the spread of this infection into the maxillary sinuses ^3^ ^,^ ^4^. In this case, it is important to use an accurate imaging study such as CBCT, which provides high-resolution images of the teeth and adjacent tissues in all planes, including the relationships between these structures ^2^ ^,^ ^3^ ^,^ ^4^.

It is necessary to emphasize the accuracy and sensitivity of CBCT compared to two-dimensional radiographs in the diagnosis of maxillary sinus changes, the assessment of the quality of endodontic treatment and the detection of periapical lesions ^2^ ^,^ ^3^ ^,^ ^4^. In the last 10 years, studies have adopted CBCT examination as the first choice for the evaluation of the paranasal sinuses and adjacent teeth ^9^. For these reasons, the CBCT scan was used to analyze the 395 teeth examined in this study, which allows a correct evaluation of the changes in the periapical tissues and maxillary sinus. The CBCT scanner used in this study was the I- CAT and the images were reconstructed with a 0.25 mm voxel ^3^ ^,^ ^12^.

Regarding the gender most affected by the inflammatory response of the maxillary sinus of odontogenic origin, our study showed that the male gender had a higher incidence, thus rejecting the null hypothesis 1, and these results are consistent with a previous study ^13^.

Some studies have reported that the maxillary first molar with a periapical lesion is the most common cause of odontogenic sinusitis, followed by the maxillary second molar ^2^ ^,^ ^14^ ^,^ ^15^ ^,^ ^16^. In this study, maxillary first and second premolars and maxillary first, second, and third molars were also selected because their roots are often located near the maxillary sinus ^3^ ^,^ ^6^. The results of the present study showed that the most affected teeth with periapical lesions were the first molars. However, there was no significant difference between the presence of an inflammatory reaction of the maxillary sinus and the location of the studied teeth ^10^ ^,^ ^17^, confirming the null hypothesis 2. The present data contrasts with previous studies ^18^ ^,^ ^19^.

The higher prevalence rates for maxillary first molars are to be expected as these teeth are more susceptible to caries, surgical procedures, and pulp inflammatory reaction of the sinus and, consequently, more vulnerable to endodontic treatment because they are the first teeth to erupt (1-4). In addition, this group of teeth has a complex internal anatomy, especially due to the fourth canal in the mesiobuccal root, which complicates treatment and increases the failure rate ^6^.

Healthy teeth whose roots are located in the maxillary sinuses can cause an inflammatory response in the sinus mucosa, and dental procedures can exacerbate the tissue response ^1^ ^,^ ^2^ ^,^ ^3^ ^,^ ^4^. In this study, the palatal root was significantly associated with the presence of maxillary sinus disease, refuting the null hypothesis 3. According to Jang et al ^20^ the palatal root of the maxillary teeth is longer than the buccal roots, which facilitates the drainage of infectious contents into the maxillary sinus. In our study, there was no correlation between the apex-cortical distance of the maxillary sinus ^21^, confirming the null hypothesis 4.

Maxillary sinusitis is associated with apical periodontitis and other odontogenic infections. Bacteria and toxins in periapical lesions may enter the maxillary sinuses directly or via lymphatic vessels and the alveolar bone marrow ^22^. The increased number of bacteria and toxins leads to greater pathogenicity of the periapical lesion, which in turn increases the likelihood of thickening of the maxillary sinus mucosa ^1^. In our study, the incidence of maxillary sinusitis in the presence of a periapical lesion was 46.6%. The most common maxillary sinus pathology was a thickening of the maxillary sinus mucosa > 3mm (59.3%), with a relatively high incidence, like the results of Aksoy & Orhan ^22^, (58.5%).

The presence of endodontically treated teeth was not significantly associated with maxillary sinus disease, in agreement with previous reports ^22^ and assuming null hypothesis 5. Mucosal thickening can be significantly affected by the presence of teeth that have undergone unsuccessful endodontic treatment as well as teeth with extensive caries and periradicular lesions ^23^. Sinusitis associated with unsuccessful endodontic treatment can be explained by the fact that irrigants, sealers, and gutta-percha may be extruded into the maxillary sinus during the procedure. Therefore long-term radiographic follow-up and clinical evaluation should be performed in these cases to monitor existing periapical and sinus pathologies.

In the study by Lu et al ^14^ a variable prevalence between 37% and 62% was found for sinus mucosal thickening associated with apical periodontitis. According to Aksoy & Orhan ^22^, this discrepancy can be attributed to the different diagnostic criteria used to assess thickening (> 1, > 2, > 3 mm). The definition of maxillary sinusitis in the different studies depends on the degree of mucosal thickening ^23^. Some studies have found that sinusitis should only be considered if the mucosal thickening is more than 2 mm ^24^. In the present study, thickening was indicative of sinusitis when it occurred at 3 mm or more ^10^.

It is important to emphasize that imaging is an additional tool for the diagnosis of sinus disease and should be based on the patient’s clinical symptoms, such as: facial pain or pressure, nasal obstruction or congestion with yellowish secretions, headache that increases in intensity with movement of the head, tenderness in the anterior region of the maxilla and infraorbital regions, eye pain, postnasal drip and bad odor ^1^ ^,^ ^3^ ^,^ ^4^ ^,^ ^25^.

It is also worth noting that histologic evaluation can accurately detect the various pathologic changes in the maxillary sinus tissue. In the present study, only database exams were analyzed without having access to the patients or their clinical records. As the study focused solely on CBCT findings, the relationship between periapical inflammatory lesions and sinus mucosal changes could not be confirmed. These results clearly indicate that future clinical studies should be carried out to evaluate the relationship between the CBCT aspect and clinical symptoms and to determine whether root canal disinfection contributes to the resolution of maxillary sinus disease.

## Conclusion

It was concluded that there is a correlation with periapical lesions and the development of maxillary sinus pathology.
